# Machine Vision-Based Quantification of Colony-Level Homing Adaptation in *Apis mellifera* Following Hive Entrance Displacement

**DOI:** 10.3390/insects17090944

**Published:** 2026-09-10

**Authors:** Run Li, Yuntao Lu, Cunchao Li, Jie Zhang, Wei Wu, Shengping Liu

**Affiliations:** Key Laboratory of Agricultural Blockchain Application, Ministry of Agriculture and Rural Affairs & Agricultural Information Institute, Chinese Academy of Agricultural Sciences, Beijing 100081, China; judy_lee_run@163.com (R.L.); 82101221354@caas.cn (Y.L.); charlesl2026@163.com (C.L.); zhangjie@caas.cn (J.Z.)

**Keywords:** *Apis mellifera*, homing, YOLO11, reorientation, temporal variation

## Abstract

Both hive displacement and entrance relocation can challenge honeybee homing navigation, yet the temporal dynamics of colony-level homing behavior following hive entrance displacement remain poorly quantified. To address this, we established a non-invasive automated pipeline integrating YOLO11m-based detection, OC-SORT tracking, and the Homing Rate (HR) to monitor nine honeybee (*Apis mellifera*) colonies under semi-natural apiary conditions. HR links trajectory endpoints with the experimentally defined valid entrance state and provides a colony-level measure of entrance-targeting accuracy. Horizontal entrance displacement caused a substantial reduction in HR in the treated colonies, whereas the Control Group showed only a small concurrent change. During the subsequent four-day observation period, all six treated colonies displayed a similar dynamic pattern characterized by an initial rapid increase in HR, followed by a slower increase. The asymptotic exponential model provided a better descriptive representation of these temporal dynamics than a linear model. The 14-day observation of Colony A1 further revealed an early increase, a transient decline, and subsequent recovery toward a relatively stable level, indicating that the post-displacement trajectory was not strictly monotonic. Overall, this study provides an automated quantitative pipeline for continuously characterizing colony-level entrance-targeting behavior and its temporal dynamics following hive entrance displacement.

## Simple Summary

Honeybees precisely navigate home, yet localized hive entrance displacement disorients homing bees. Most studies focus on long-range navigational cues, while the continuous quantification of short-range reorientation dynamics remains constrained by technical bottlenecks. In this study, we monitored nine honeybee (*Apis mellifera*) colonies and automatically tracked returning bees using time-series video data. Homing Rate (HR) was calculated by linking trajectory endpoints with entrance states to quantify colony-level entrance-targeting accuracy. Following horizontal entrance displacement, HR declined substantially in the treated colonies, whereas only a small concurrent decline occurred in the control colonies. During the four days following displacement, all six treated colonies showed a similar pattern, with an initial rapid increase in HR followed by a slower increase. The extended 14-day observation of Colony A1 further showed an early increase, a transient decline, and a subsequent approach toward a stable level below 100%. These findings show that automated trajectory analysis can quantitatively characterize colony-level short-range homing reorientation following local entrance displacement.

## 1. Introduction

Honeybees have long been used to study insect navigation mechanisms, particularly homing behavior [1]. A bee’s ability to return to the hive is driven by a combination of genetic predisposition and acquired learning experience [1,2,3]. It is widely believed that bees construct a spatial memory template centered on the hive through learning flights [4,5,6], which subsequently guides their homing behavior. Bee navigation involves various cues [3,7] such as vision [5,7,8], solar position and polarized light [6,9,10], the geomagnetic field [8,11,12], and pheromones [13,14,15]. Although the interactions and relative contributions of these cues remain a mystery, the poor homing performance of bees during a total solar eclipse demonstrates that visual information remains an indispensable basis for navigation even under extreme conditions of very low light [16]. Research on the influence of visual information has revealed that bee navigation exhibits a hierarchical spatial structure [3]. During long-range orientation, bees rely on panoramic visual cues such as ridges or forests to locate the broad area of the hive [17,18]. In contrast, the precise identification of the hive entrance during short-range orientation is highly dependent on local visual features such as hive color, nearby rocks, or artificial geometric markers [7,19,20,21]. This strong reliance on local cues is particularly evident in experiments with digger wasps, where the displacement of visual markers causes a synchronous shift of the wasps’ localization point to the new position [19]. Von Frisch found similar evidence of visual landmark use in honeybees, displacing colorful panels from around the hive with homing bees following these displaced landmarks to find the hive [21].

Existing studies have primarily quantified the impact of informational cues on honeybee homing via three experimental paradigms: (a) releasing marked bees at unfamiliar sites [3,5,7,9,10,11,12,16,17,18], (b) changing or moving the hive landmarks [7,19,20,21], and (c) displacing the hive [22,23,24]. These changes alter the external visual cues during bees’ homing, while neglecting the impact of changing the hive entrance location on the normal homing of honeybee colonies. This issue corresponds to double hive doors, hive door adjustments, and intelligent hive entrance management in beekeeping practice. Such displacement may occur in beekeeping practices with two-entrance hives and cause confusion [25,26]. When the physical location of the entrance changes abruptly while surrounding local visual cues remain unchanged, bees experience a conflict between their visual memories of the original entrance location and the displaced entrance location.

Effectively and continuously quantifying changes in honeybee homing behavior following hive entrance displacement under natural apiary conditions poses a methodological gap. Traditionally, honeybee homing studies relied on manual tagging [16,19,27], RFID tagging [9,28], or harmonic radar tracking [3,17,29,30]. Manual observation is labor-intensive and error-prone for long-term monitoring. RFID transponders cannot capture spatial trajectories. Harmonic radars are cost-prohibitive and introduce heavy physical loads that alter natural behavior. Meanwhile, high-resolution 3D reconstruction provides precise paths [11,31,32]. Its extreme computational complexity and strict laboratory setups render long-term apiary deployment unfeasible. Recent computer vision studies in apiculture mostly focused on static detection, behavior classification, or entrance counting [33,34,35,36,37]. However, entrance activity counts and spatial distributions alone cannot directly indicate whether homing honeybees target the currently valid or original entrance. Continuous trajectory information needs to be linked with entrance states to quantify entrance-targeting outcomes. To address this gap, this study assembles a non-invasive, high-throughput digital phenotyping pipeline integrating S6YOLO11m and OC-SORT to continuously monitor honeybee homing behavior under localized hive entrance disturbances. Within this pipeline, Homing Rate (HR) was introduced as a quantitative metric of entrance-targeting accuracy, enabling temporal analysis of colony-level homing behavior.

## 2. Materials and Methods

**Figure 1:**
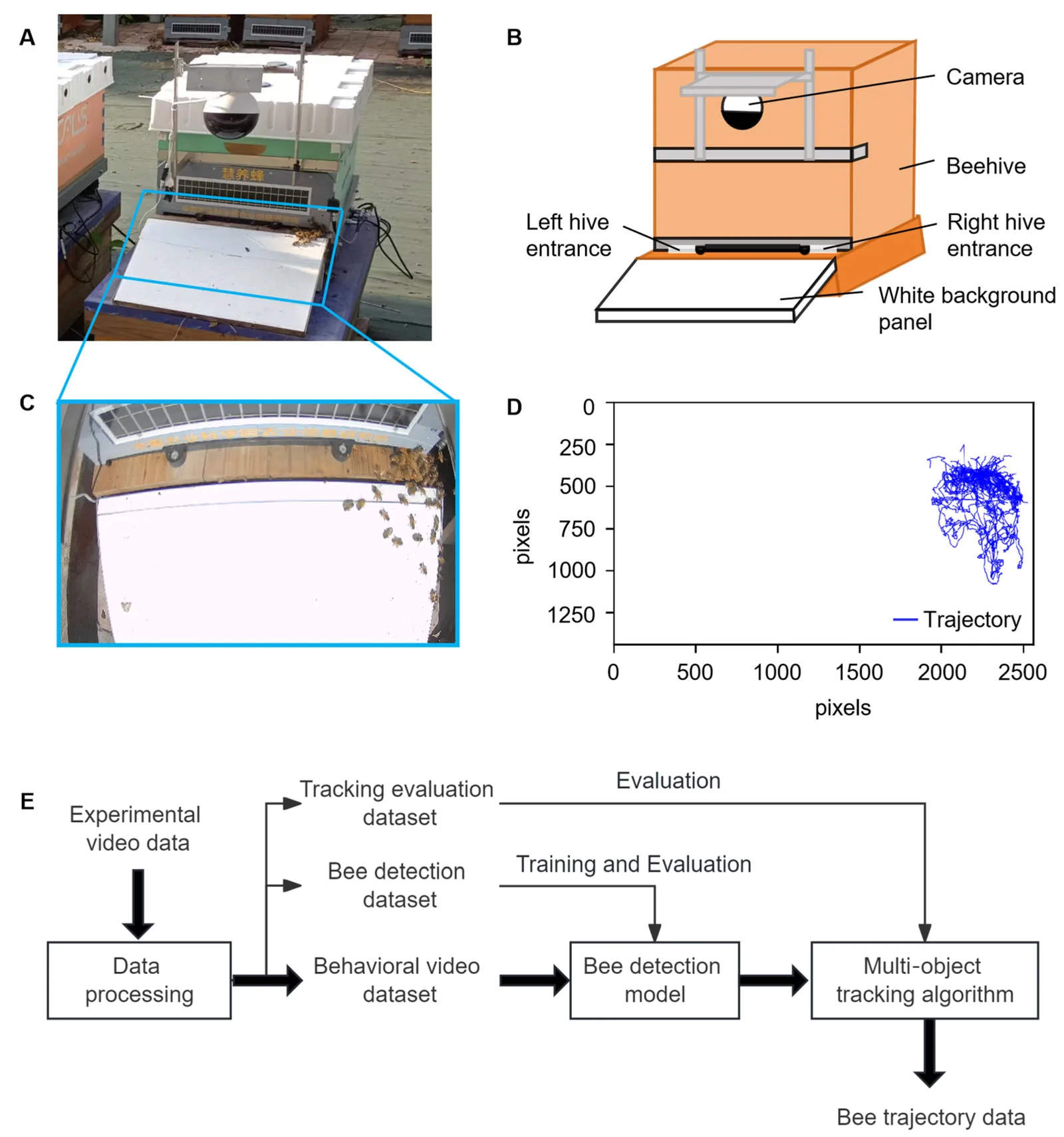
Representation of the video recording setup. The figure shows a side view of the experimental setup (**A**) and a schematic diagram of the experimental setup (**B**). A representative frame from the monitoring video is shown in (**C**). The YOLO11m detection model and OC-SORT were then applied to track the trajectories of these bees (**D**). The overall data processing workflow is illustrated in (**E**). The Chinese text on the hives in panels (**A**,**C**) identifies the Agricultural Information Institute, Chinese Academy of Agricultural Sciences, where the hives were custom-built.

This section provides a detailed description of the experimental setup, video acquisition, and processing procedures used to study honeybee homing behavior under varying hive entrance locations and introduces the algorithms used to detect and track bees. Figure 1 provides a comprehensive overview of the entire experimental setup and the video data acquisition and processing workflow. Finally, the text provides a detailed explanation of the boundary criteria for trajectory direction classification and region segmentation, the formula for calculating HR, as well as the statistical analysis methods and computational platforms used to evaluate the patterns of colony behavior evolution.

### 2.1. Materials and Experimental Setup

The hive entrance displacement experiments in this study were conducted at the Nankou Station of the Chinese Academy of Agricultural Sciences, Changping District, Beijing, China (116.321° E, 39.959° N) from 1 August to 14 September 2025. Nine healthy colonies of honeybees (*Apis mellifera*) were utilized, each housed in a separate hive spaced 10 cm apart. Hive placement was randomized across the apiary, as shown in Supplementary Figure S1. These hives were placed in a controlled apiary environment under comparable conditions (temperature: 28.38 ± 5.57 °C; relative humidity: 81.22 ± 12.62%). Temperature and relative humidity were recorded by the smart hive sensor system developed at the Agricultural Information Institute, CAAS (Beijing, China). All colonies were headed by two-year-old queens, maintained a colony strength of 4–5 frames (approximately 9000–12,000 worker bees), received no artificial feeding during the experimental period, and were confirmed free from Varroa destructor mites and other common diseases.

To achieve monitoring of homing behavior, a custom two-entrance structure was adopted for each hive. Before the experiment, only a single entrance was open to allow the bees to habituate to the initial location. A white background panel was mounted at an angle in front of each entrance apron to enhance image contrast, thereby improving the identification accuracy of dense-bee targets [38]. The EZVIZ C8W cameras (Hangzhou EZVIZ Software Co., Ltd., Hangzhou, China) were mounted 40 cm directly above the entrance of each hive at the same top-down angle, with their fields of view fully covering both hive entrances and the landing apron area to continuously monitor homing behavior. Detailed specifications of the hive and monitoring equipment are provided in Supplementary Table S1. Nine honeybee colonies were divided into three groups to estimate confounding effects caused by lateral preference (Table 1). These three groups were Right-shifted Group (entrance right-shifted horizontally 30 cm; colonies A1–A3), Left-shifted Group (entrance left-shifted horizontally 30 cm; colonies B1–B3), and Control Group (unshifted; colonies C1–C3) [39].

The experimental timeline comprised a 2-day baseline period (1–2 August) and a 2-day post-displacement adjustment period (1–2 September). The hive entrance displacement was executed at 06:00 on 1 September 2025, aligning with local sunrise to minimize disruption to the colonies’ natural foraging rhythm, as *A. mellifera* homing activity intensifies rapidly post-sunrise [40]. Video recording was conducted daily from 06:30 to 18:30, yielding a continuous 12-h daytime monitoring window per day. Although the baseline period and post-displacement period were separated by a one-month interval and accompanied by potential changes in environmental factors such as seasonal light, weather, and local nectar sources, all experimental groups were exposed to these concurrent environmental changes. Therefore, the synchronous Control Group was included to provide a reference for background temporal variation.

### 2.2. Video Data Processing

To train the computer vision models separately from the behavioral evaluation, reference video sequences of 11 independent honeybee colonies were recorded from August to October 2024. These videos were strictly separated by time and origin from the 2025 experimental videos to maintain independence between model development and the behavioral experiment. From those raw videos, three core datasets were constructed to analyze honeybee activities at hive entrances [37], namely the bee detection dataset, the tracking evaluation dataset, and the behavioral video dataset. The comprehensive workflow for data processing and behavioral analysis is illustrated in Figure 1E. To efficiently quantify collective insect activities under intense mutual occlusion at the semi-natural hive entrance, Tracking-by-Detection (TBD) was implemented. This paradigm purposefully decouples single-frame spatial detection from cross-frame temporal association, mitigating identity switches during prolonged crowding while ensuring stable and predictable computational demands when scaling up to large-scale behavioral datasets.

#### 2.2.1. Bee Detection Dataset for Model Training

**Figure 2:**
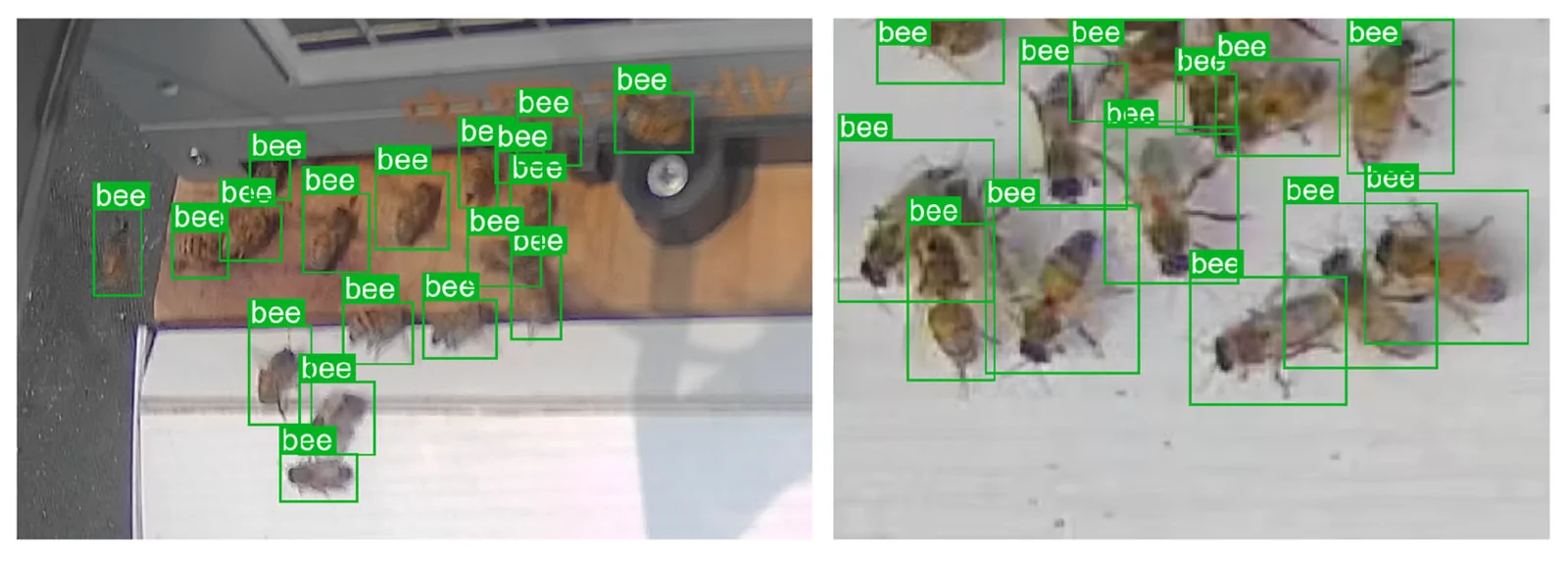
Samples of bee detection dataset.

To construct the bee detection dataset, we performed stratified random sampling on the raw daytime videos (August to October 2024), extracting frames from each hour at intervals of 200 frames to maintain balanced temporal representation. Typical honeybee poses involving flying, crawling, and mutually obscured individuals were annotated using the X-AnyLabeling (https://github.com/CVHub520/X-AnyLabeling, accessed on 2 December 2024), yielding 22,529 valid bounding boxes across 1003 original images, as illustrated in Figure 2.

The annotated images were randomly partitioned into training, validation, and testing subsets at percentages of 64%, 16%, and 20%, respectively. To enrich training diversity, offline data augmentation, including horizontal flipping, random brightness or contrast adjustments, and small-angle rotations, was applied exclusively to the training subset, expanding it from 641 to 1282 images. The validation and testing subsets remained unmodified, resulting in a final dataset of 1644 images saved in the standard YOLO format [35]. The dataset spans a broad illumination range matching diurnal apiary profiles. See Supplementary Table S2 for full grayscale distribution details. To further verify whether this compiled dataset aligns with the illumination intensities across the monitoring period, the mean grayscale intensity of each image was quantified on a scale from 0 to 255. As summarized in Table S2, the overall illumination intensity (grayscale) ranged from 54.51 to 216.14 (mean: 143.28 ± 20.75), which closely conformed to the diurnal light profiles of the observation window. Based on this dataset, transfer learning was applied across the full spectrum of YOLO11 configurations (YOLO11n, s, m, l, and x) to establish an optimized detector. YOLO11 was selected due to its architectural optimizations, enhanced C3k2 and SPPF modules, which are good at extracting fine-grained features of high-velocity, small-scale targets across multi-oriented bee poses, such as flying, crawling, and hovering [37,41,42]. It is suitable for complex agricultural field environments involving variable illumination, occlusion, and multiscale targets [43,44]. Evaluating these five scales serves the purpose of systematically identifying the optimal convergence point between bounding-box localization precision and computational inference speed, which is a prerequisite for analyzing millions of continuous multi-day video frames.

#### 2.2.2. Tracking Evaluation Dataset for MOT Algorithm Selection

The tracking evaluation dataset consisted of four 10-s video clips extracted from the raw videos recorded in September 2024. This 40-s evaluation sequence contained a total of 10,224 manually annotated bounding boxes for comparative evaluation of the candidate tracking algorithms. As detailed in Table 2, the dataset exhibits average honeybee densities ranging from 6.1 to 16.4 bees/frame. Honeybee trajectories and unique identification tags were manually annotated frame-by-frame to establish the ground truth for multi-object tracking evaluation [37]. Under the Tracking-by-Detection paradigm, four representative tracking algorithms, including DeepSORT [41], StrongSORT [42], ByteTrack [45], and OC-SORT [46], were selected for comparative evaluation to explore how different technical mechanisms optimize trajectory continuity under biological constraints. Detailed technical mechanisms and theoretical principles of these tracking algorithms are provided in Supplementary Note S1. These clips were recorded under the same fixed-camera hive-entrance monitoring configuration used in the subsequent experiment and were used to support comparative tracker selection rather than to evaluate cross-year tracking generalization. Testing these diverse multi-object tracking algorithms on the tracking dataset provides an empirical basis for selecting the most suitable tracking algorithm to extract subsequent trajectory data.

#### 2.2.3. Behavioral Video Dataset for Ethological Analysis

To capture diurnal homing behavior, continuous video recordings were conducted for each experimental colony from 6:30 to 18:30 daily, accumulating 12 h of raw videos per colony per day. Adopting the scan sampling method [47,48], One-minute segments were extracted at each full hour (i.e., from 7:00 to 18:00 at 12 discrete time points), resulting in a total of 12 min of representative sample per colony per day [34]. The experiment involved nine colonies divided into three treatment groups with three replicates each. The data volume for each Right-shifted Group across all experimental periods is summarized in Table 3. The dataset was established across four chronological periods to capture different behavioral contexts. First, the Baseline dataset was collected under natural conditions to serve as a control. Second, the Adjustment dataset was acquired during the hive entrance displacement intervention. To further analyze the sequential dynamics following the intervention, the Post-intervention dataset tracked six colonies from Groups A and B. Finally, Colony A1 was monitored for an extended period to construct the Long-term tracking dataset for long-term adaptation assessment. Before extracting behavioral matrices from these multi-period datasets, video frames were horizontally calibrated [49]. Using the hive entrance edge as the baseline, the frames were adjusted so that the hive was positioned strictly above the center of the video, with the hive edge parallel to the horizontal axis. Time-series trajectory data were subsequently extracted from this calibrated dataset using the bee detection and tracking model [35].

### 2.3. Quantification of Honeybee Homing Behavior

#### 2.3.1. Trajectory Preprocessing and Direction Classification

Before behavioral quantification, fragmented short trajectories (duration < 10 frames) and trajectories containing abnormal frame-to-frame displacement jumps (>100 pixels) [50] were excluded to reduce the contribution of clearly incomplete or discontinuous tracks to subsequent trajectory-based behavioral analyses. Based on this filtered dataset, the trajectories were categorized to facilitate distinct analytical approaches. First, the homing-intent trajectory was defined as paths moving upward toward the hive at the top of the frame. In the standard image coordinate system with a top-left origin, this upward movement was quantified by an endpoint y-coordinate that was less than the starting point y-coordinate. This specific subset served as the data foundation for subsequent visualization analyses, including heatmaps and trajectory plots. The heatmap was generated by applying a two-dimensional kernel density estimation (KDE) with Scott’s bandwidth rule to all tracked coordinate points. The resulting density data were smoothed using a Gaussian filter ( σ = 2) and normalized to a relative scale. Second, a conceptual classification of trajectory directions was established based on the vector angle (detailed in Section 2.3.2), which was utilized solely for the descriptive visualization of trajectory plots. Finally, homing trajectories were defined according to spatial criteria relative to the near-entrance area of the hive [5] (detailed in Section 2.3.3).

#### 2.3.2. Trajectory Direction Classification

Building upon the homing-intent trajectories defined in Section 2.3.1, this study further classified and visualized trajectory directions. To intuitively present the spatial distribution of trajectory directions, continuous angular data were discretized into four quadrants for descriptive summaries (Table 4). Detailed mathematical formulations for Cartesian coordinate system establishment and trajectory angle calculation are provided in Supplementary Note S2. Notably, the Upward category is not a parallel concept to homing intent, but rather a subset representing a direct radial movement toward the hive within the homing-intent trajectories, thereby forming a part-to-whole relationship. This directional classification serves exclusively as a qualitative visualization tool to map the spatial distribution of different directional categories in trajectory plots, and it does not involve the calculation of any quantitative metrics.

#### 2.3.3. Spatial Zoning and Homing Rate Calculation

**Figure 3:**
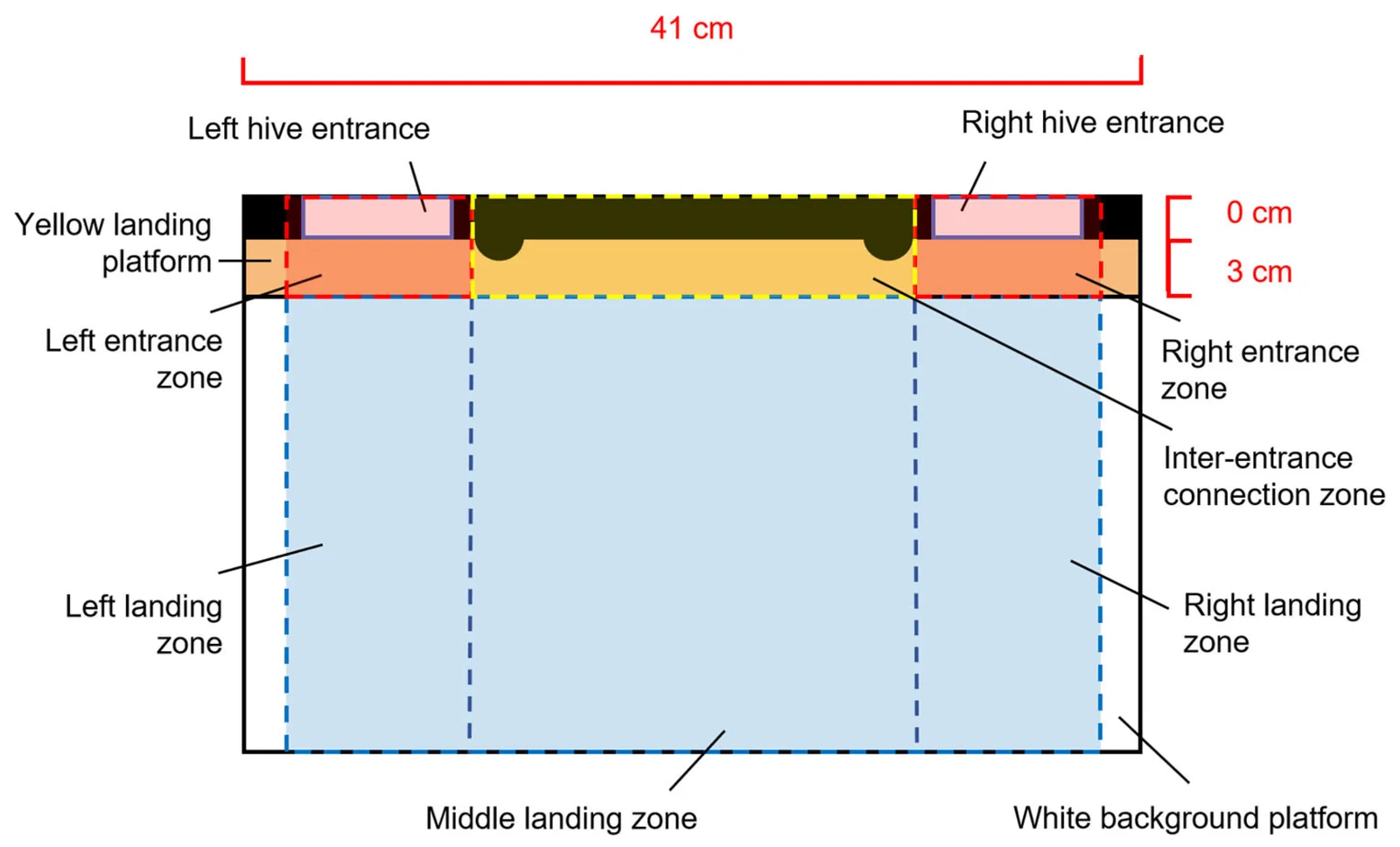
Schematic diagram of spatial zoning and boundary calibration for the monitoring area. Red dashed boxes, hive entrance tracking zones (6 cm × 3 cm, defined by extending the physical opening by 0.5 cm); yellow dashed box, inter-entrance connection zone (3 cm width); blue dashed box, landing zone. The red scale bar indicates the baseline calibration dimensions (41 cm × 3 cm).

Based on the filtered homing trajectories from Section 2.3.1, this study implemented spatial zoning to calculate the HR. According to the structure of the beehive, the monitored area was spatially partitioned into six functional zones (Figure 3). Horizontally, it comprised the left, transition, and right zones; vertically, it was divided into the hive entrance zone and the front platform zone. The motion coordinates of the trajectories were mapped to these categories to describe the spatial distribution preferences [50]. For quantitative behavioral analysis, the pixels were converted into physical distances (centimeters). The spatial calibration was locally established based on the yellow landing platform (Figure 3), which served as a reliable and planar geometric reference. The scale conversion factors for the horizontal and vertical axes were independently calibrated as 0.0252 cm/pixel and 0.0246 cm/pixel, demonstrating a highly consistent pixel-to-physical ratio between the horizontal and vertical axes in the yellow landing platform.

Unlike conventional long-distance HR that measures homing success from remote release sites, the HR defined in this study quantifies the accuracy of entrance targeting during the final stage of homing. The box method was used at the hive entrance zone (the red dashed boxes in Figure 3) to determine whether a honeybee entered the entrance zone [37]. The Homing Rate (HR) is defined as the ratio of the number of bee trajectories successfully entering the currently open (valid) hive entrance to the total number of trajectories entering the designated regions on both sides of the hive entrances. The HR metric is formulated as follows:

(1) $$\mathrm{Homing}\;\mathrm{Rate}\;=\;\frac{N_{\mathrm{valid}}}{N_{\mathrm{left}}\;+\;N_{\mathrm{right}}}$$

Here, $N_{\mathrm{left}}$ and $N_{\mathrm{right}}$ denote the total number of trajectories whose endpoints were located respectively within the left and right hive entrance zones, strictly requiring their starting points to be outside these zones [34,38]. $N_{\mathrm{valid}}$ represents the number of trajectories successfully entering the valid hive entrance area where the hive gate is actually open. The conditional logic is as follows:

(2) $$N_{\mathrm{valid}}\;=\;\left\{\begin{array}{c} {\;N}_{\mathrm{left}},\;If\;the\;left\;entrance\;is\;open,\\ \;N_{\mathrm{right}},\;If\;the\;right\;entrance\;is\;open. \end{array}\right.$$

It is important that the algorithm relies on stable cross-frame target tracking IDs to prevent duplicate counting. A valid homing event is recorded only when a unique trajectory ID meets the strict spatial criteria (starting outside the zone and ending inside). If a honeybee hovers, enters the zone, and then flies away without staying, its trajectory endpoint will eventually fall outside the zone, thus failing the termination constraint and not being counted. This start-outside-and-end-inside trajectory logic effectively prevents the double-counting of individual bees repeatedly visiting or loitering at the entrance.

### 2.4. Statistical Testing

Method agreement between automated and manual HR measurements was evaluated using linear regression, the coefficient of determination ( $R^{2}$ ), root mean square error (RMSE), mean bias (MB), and Bland–Altman analysis with 95% limits of agreement. Measurement differences were calculated as automated HR minus manual HR, where a positive MB indicated overestimation by the automated method. Pearson’s correlation coefficient (*r*) was used to evaluate the linear relationship between the two HR. The validation was conducted separately for the baseline and adjustment periods. For the baseline period, 12 paired HR observations were obtained from six treated colonies, with two observations per colony. For adjustment-period validation, 24 paired HR observations were collected from the same six colonies at two daily time points on Days 1 and 4 post-displacement. All observations were obtained from the 2025 experimental videos used for behavioral analysis.

Rate difference (RD) in homing rate (HR) was used as the primary effect measure of hive entrance displacement. For between-period comparisons within each group (Right-shifted, Left-shifted, and Control), RD was calculated to characterize the direction, magnitude, and consistency of temporal changes. For between-group comparisons within each period(baseline and adjustment), RD was calculated to assess baseline and adjustment group differences. As a complementary analysis, the binomial generalized linear mixed model (GLMM) with a logit link was fitted to aggregated success and failure homing outcomes for each colony and period. Group, period, and their interaction were included as fixed effects, with colony identity as a random intercept to account for the non-independence of repeated measurements within colonies. Fixed effects were evaluated using Type III Wald $\chi^{2}$ tests, followed by Tukey-adjusted pairwise comparisons. Given the limited number of independent colonies (*n* = 3 per group), GLMM results were interpreted as complementary statistical support for the RD in colony-level HR values.

The dynamic synchrony of HR time series was evaluated using Pearson’s correlation coefficient [39]. Within-group consistency was measured by correlating individual colony HR series with the mean series of their respective experimental group, while between-group consistency was measured by correlating the mean series of the two groups. We used the asymptotic exponential model from behavioral ecology [50,51,52,53] and compared it with a linear model to describe the daily temporal trends in colony-level HR over the 4-day period following nest entrance displacement. The daily HRs from six treated colonies were summarized and fitted using the two mixed-effects models, with colony identity included as a random effect to account for repeated measurements. The asymptotic exponential model is defined as:

(3) $$y(t)=a\;\cdot \;(1\;-\;e^{-kt}),$$

where *y*(*t*) denotes the HR on Day *t*, a is the asymptotic limit, and *k* is the rate constant. Both models were fitted via maximum likelihood estimation and evaluated using the small-sample corrected Akaike Information Criterion (AICc). Colony-level asymptotic models were additionally fitted using weighted nonlinear least squares (WNLS). Proportional weights $w\;=\;\frac{1}{p\;(1-p)}$ were applied to reduce heteroscedasticity in proportional data, where *p* denotes the daily mean HR. All model-based analyses were conducted in R version 4.3.1.

### 2.5. Data Analysis and Computing Environment

All data processing, model training, and metric quantification were implemented using Python (v3.10). Image processing, tracking inference, and metric quantification were executed in Python using OpenCV (v4.10.0), pandas (v2.2.3), scikit-learn (v1.6.1), and motmetrics (v1.4.0). Statistical tests were performed using SciPy (v1.13.1) and Statsmodels (v0.14.5), except for those explicitly conducted using R as specified in Section 2.4. Pipelines were deployed on a workstation equipped with an NVIDIA GeForce RTX 4090 GPU (NVIDIA Corporation, Santa Clara, CA, USA) with 24 GB VRAM. All statistical tests were two-tailed with a significance threshold of *α* = 0.05.

## 3. Results

### 3.1. Performance of Honeybee Detection and Tracking Models

The evaluation results are summarized in Table 5 and Table 6, where the best and second-best performances for each metric are highlighted in bold and underlined. Evaluation of the five YOLO11 models on the bee detection dataset showed that YOLO11m achieved the optimal balance between accuracy and efficiency. It yielded a Precision of 91.6 and an mAP@0.5:0.95 of 57.2 on the test set, while maintaining a high processing speed of 27.91 fps (detection examples are shown in Figure 1C). Regarding multi-object tracking in the monitoring area, OC-SORT and ByteTrack showed different performance advantages. OC-SORT achieved the highest MOTA (67.2) and MOTP (72.9), the second-highest IDF1 (64.2), and produced substantially fewer FPs (278). ByteTrack yielded the highest IDF1 (68.5), the lowest IDsw (8) and FN (243), but produced substantially more FP (1167). Based on these benchmark results, the combined YOLO11m and OC-SORT pipeline was selected to extract the subsequent honeybee trajectory data.

### 3.2. Validation of Automated Homing Rate Measurement

**Figure 4:**
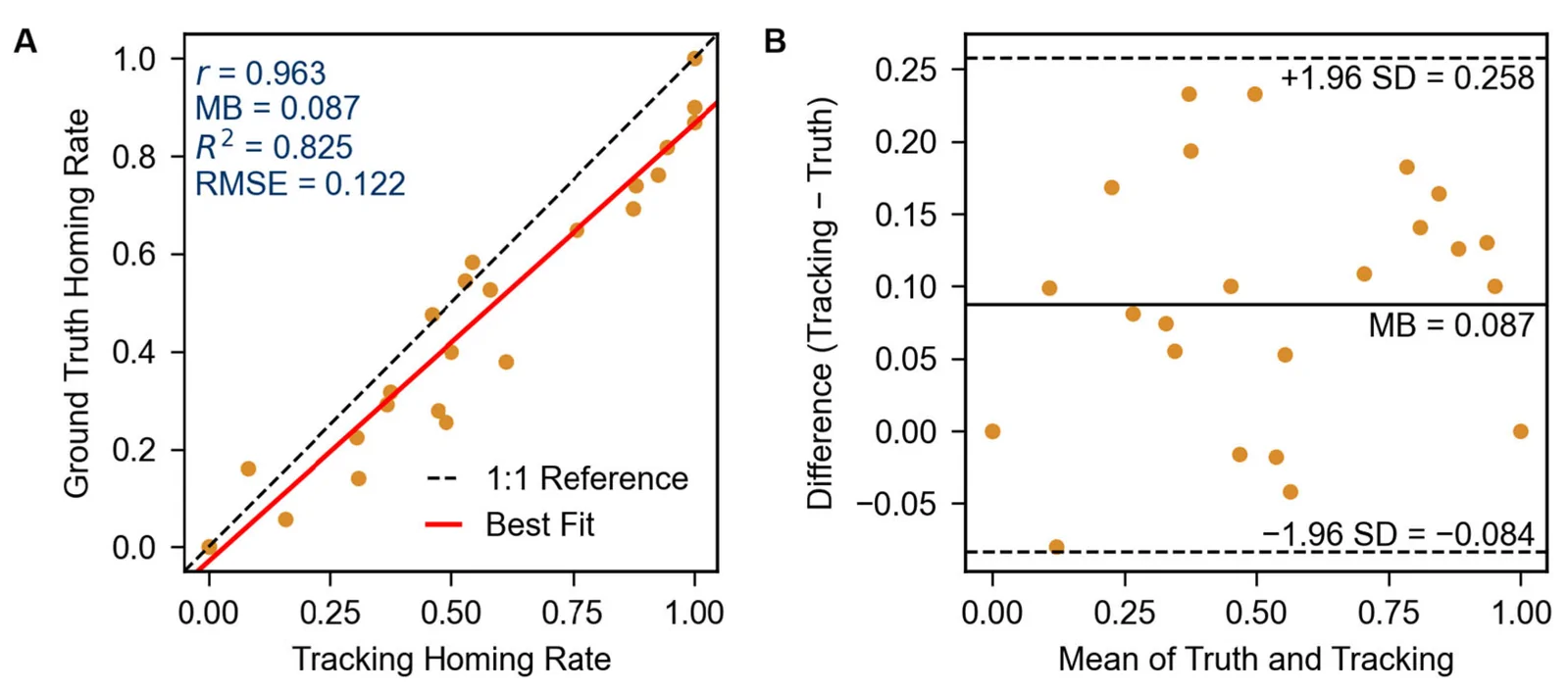
Validation of the automated Homing Rate during the adjustment period. (**A**) Relationship between automated and manually counted HR across six treated colonies. The solid line is the fitted linear regression, and the dashed line indicates the line of identity (y = x). (**B**) Bland–Altman plot of the difference between automated and manual measurements against their mean. The solid horizontal line represents the mean bias, and the dashed lines show the 95% limits of agreement.

Before assessing the robustness of the HR, it is crucial to clarify its spatial scope in this study. Here, HR is strictly defined as the proportion of bees successfully targeting the correct entrance within the near-entrance area of the hive, rather than the long-range homing success typically measured over kilometers. To evaluate measurement agreement, automated HRs were compared with manual observations. Period-level validation showed different levels of measurement error between the baseline and adjustment periods (Table 7). During the baseline period, the $R^{2}$ between automated and manual HR was 0.780, while the measurement error was small (RMSE = 0.010, MB = −0.002), with 95% limits of agreement from −0.022 to 0.017. During the adjustment period, linear regression showed a strong correlation (*r* = 0.963, $R^{2}$ = 0.825; Figure 4A). Bland–Altman analysis revealed a systematic overestimation by the automated method (MB = 0.087, RMSE = 0.122), with 95% limits of agreement ranging from −0.084 to 0.258 (Figure 4B). Thus, measurement error was greater during the adjustment period than during the baseline.

### 3.3. Effect of Hive Entrance Displacement on Homing Behavior of Honeybee Colonies

#### 3.3.1. Heatmap Analysis

**Figure 5:**
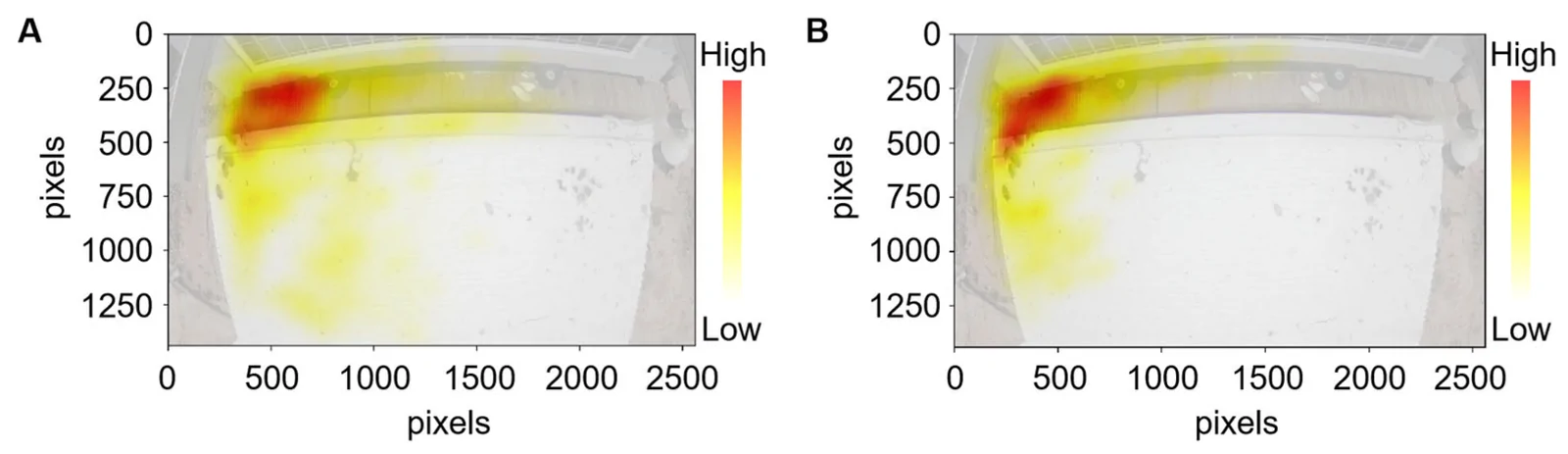
Comparison of hive entrance heatmaps of the colony C1 (Control Group, not shifted) between the baseline and adjustment periods. (**A**) Second day of the baseline period. (**B**) First day of the adjustment period. The heatmap represents the continuous two-dimensional kernel density estimation (KDE) calculated from the coordinate positions of tracking trajectories. The color scale transitions from white (zero density), through yellow (low relative density), to red (high relative density), indicating the relative dwell intensity of bees. The density estimation utilizes an automatic bandwidth determination via Scott’s rule, smoothed by a Gaussian filter ( σ = 2).

To assess potential temporal effects between experimental periods, hive entrance activity heatmaps from the control colony C1 were compared. As shown in Figure 5, no obvious differences were observed in the spatial distribution between the baseline and adjustment periods.

**Figure 6:**
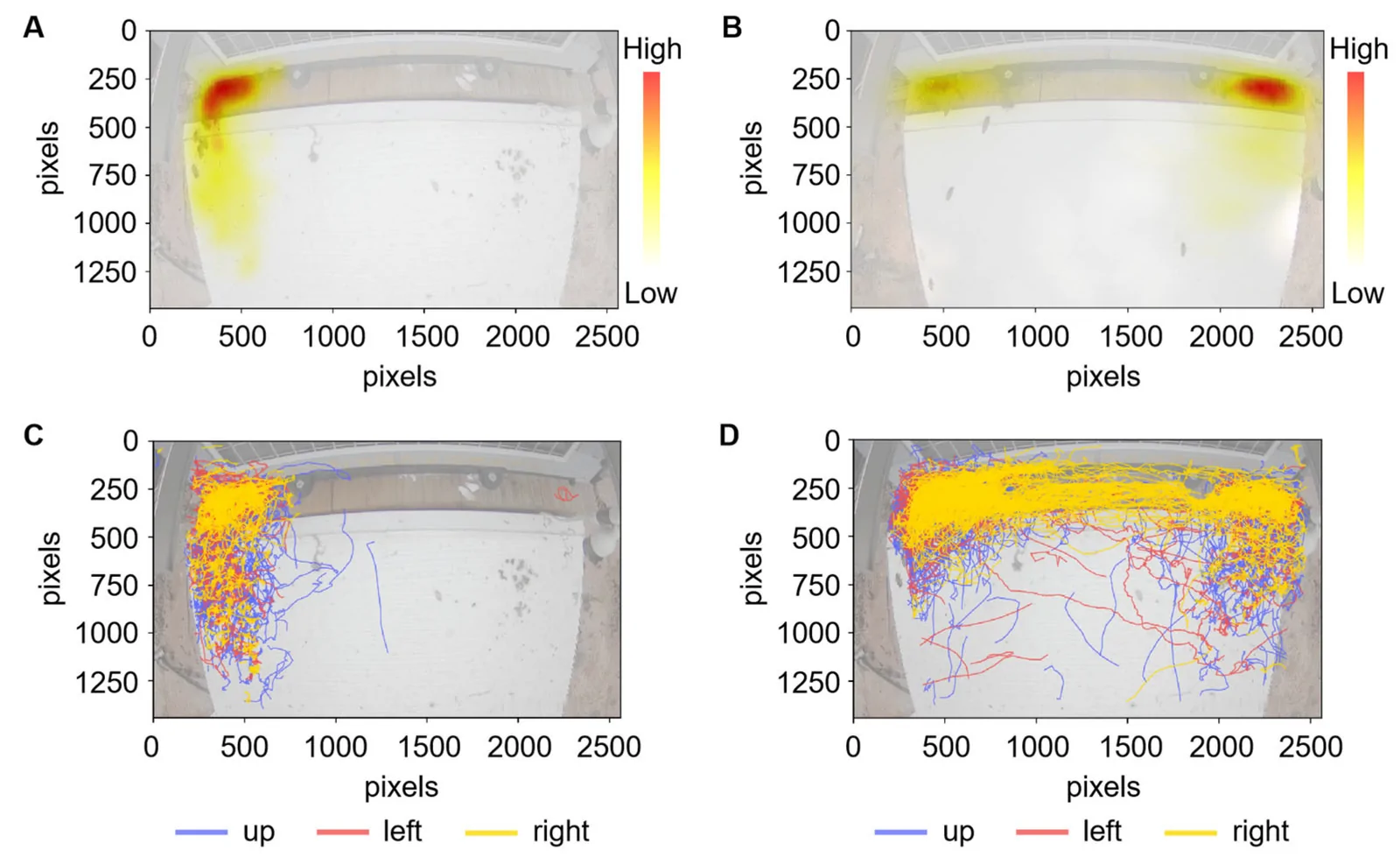
Comparison of heatmap and trajectory patterns of colony A1 (Right-shifted Group) before and after displacement. (**A**) Activity heatmap on the second day of the baseline period. (**B**) Activity heatmap on the first day of the adjustment period. (**C**) Directional trajectory pattern on the second day of the baseline period. (**D**) Directional trajectory pattern on the first day of the adjustment period. The heatmap is generated and visualized using the identical methods described in Figure 5.

For the displacement treatment, during the baseline period, the activity of Colony A1 was concentrated around the left open entrance and its corresponding landing area, exhibiting a single-center aggregation pattern (Figure 6A). Upon entering the adjustment period with the entrance shifted to the right, the heatmap transitioned to a bimodal distribution, characterized by behavioral aggregation at both the new right entrance and the original left entrance location (Figure 6B).

#### 3.3.2. Direction-Specific Trajectory Analysis

During the baseline period, the upward, leftward, and rightward trajectories were highly overlapped in space (Figure 6C). In the adjustment period, the planar spatial distributions of these three trajectory types became separated. The upward and leftward trajectories were distributed over both entrances and the apron areas, whereas the rightward trajectories covered both entrances and the connection area between them (Figure 6D).

#### 3.3.3. Significant Difference in Homing Rate

**Figure 7:**
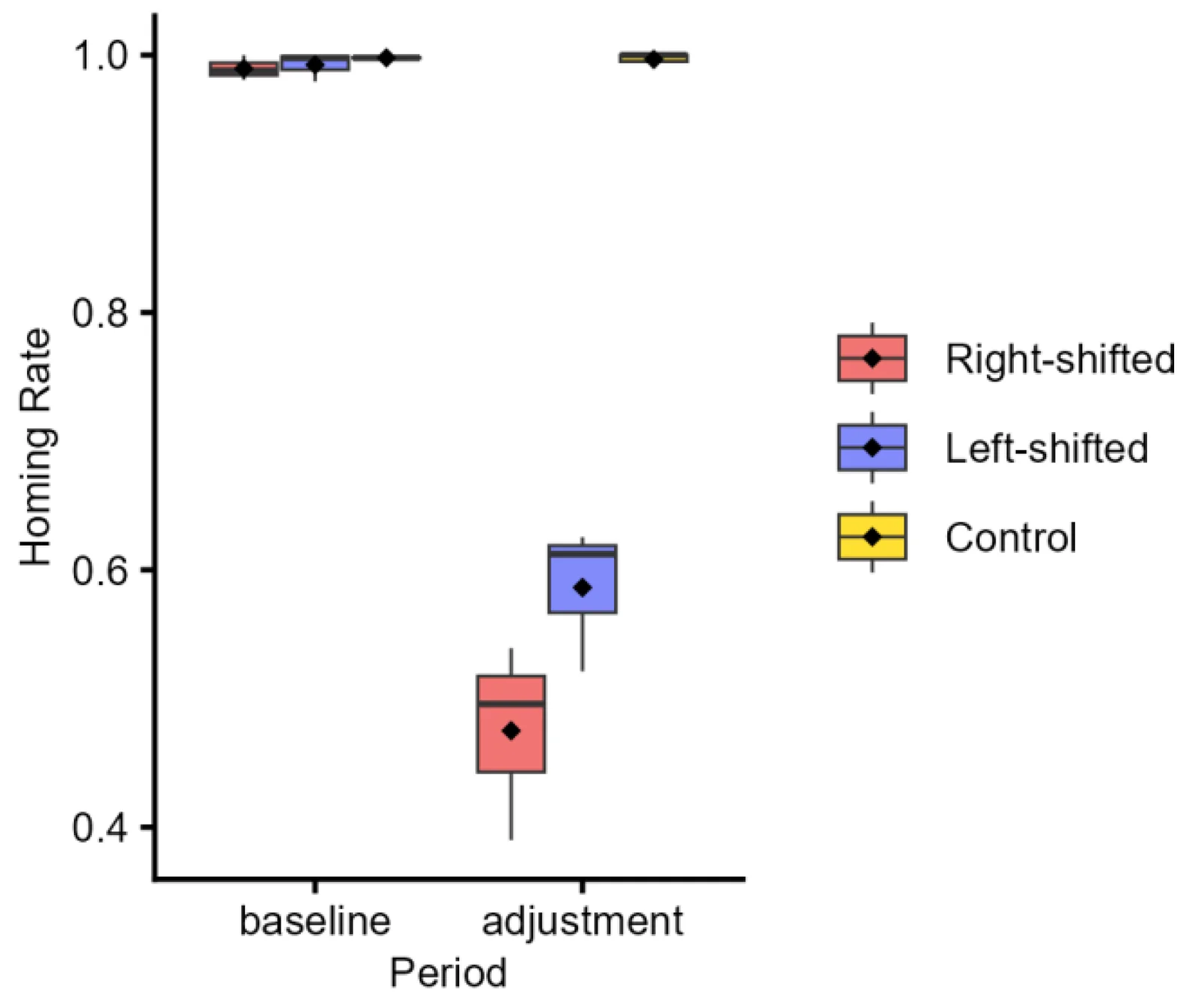
Comparison of Homing Rate across groups and periods. Black horizontal lines indicate medians; diamonds indicate means; boxes represent the interquartile range (IQR); whiskers extend to 1.5 × IQR. Data are shown for the Right-shifted, Left-shifted, and Control groups during the baseline and adjustment periods (*n* = 3 colonies per group per period).

The HR across the three groups during the baseline and adjustment periods is illustrated in Figure 7. During the baseline period, HR remained consistently high across all groups, exceeding 99%, with only small between-group differences. Following entrance displacement, HR decreased markedly in the Right-shifted and Left-shifted groups, whereas the Control group showed a slight decline. The direction of change was consistent among the three colonies within each treated group. Consistent with these colony-level HR patterns, the GLMM showed an interaction between Group and Period (Table 8; χ^2^ = 42.12, *p* < 0.001) in the present dataset.

RD (rate difference) was used to quantify the magnitude of these HR changes. Between-period comparisons were performed to evaluate changes in HR within each group (Table 9). For the Control group, the HR showed slight decline between periods (RD = −0.13%, *p* = 0.448). In contrast, HR decreased by 50.36% in the Right-shifted group and by 40.89% in the Left-shifted group relative to their respective baseline levels (both *p* < 0.001). Between-group RDs were then compared within each period (Table 10). During the baseline period, Right-shifted (RD = −0.68%) and Left-shifted (RD = −0.53%) HR declined slightly from Control group. During the adjustment period, HR dropped by 50.91% in the Right-shifted group and 41.29% in the Left-shifted group compared to the Control group (both *p* < 0.001). The RD between the Right-shifted and Left-shifted groups was −9.62% (*p* = 0.015). The complementary GLMM-based pairwise comparisons were generally consistent with these RD patterns. The corresponding ORs and 95% CIs are reported in Table 9 and Table 10.

### 3.4. Adaptation Dynamics of Honeybee Colonies Based on Homing Rate

#### 3.4.1. Time-Series Analysis of HR Dynamics

**Figure 8:**
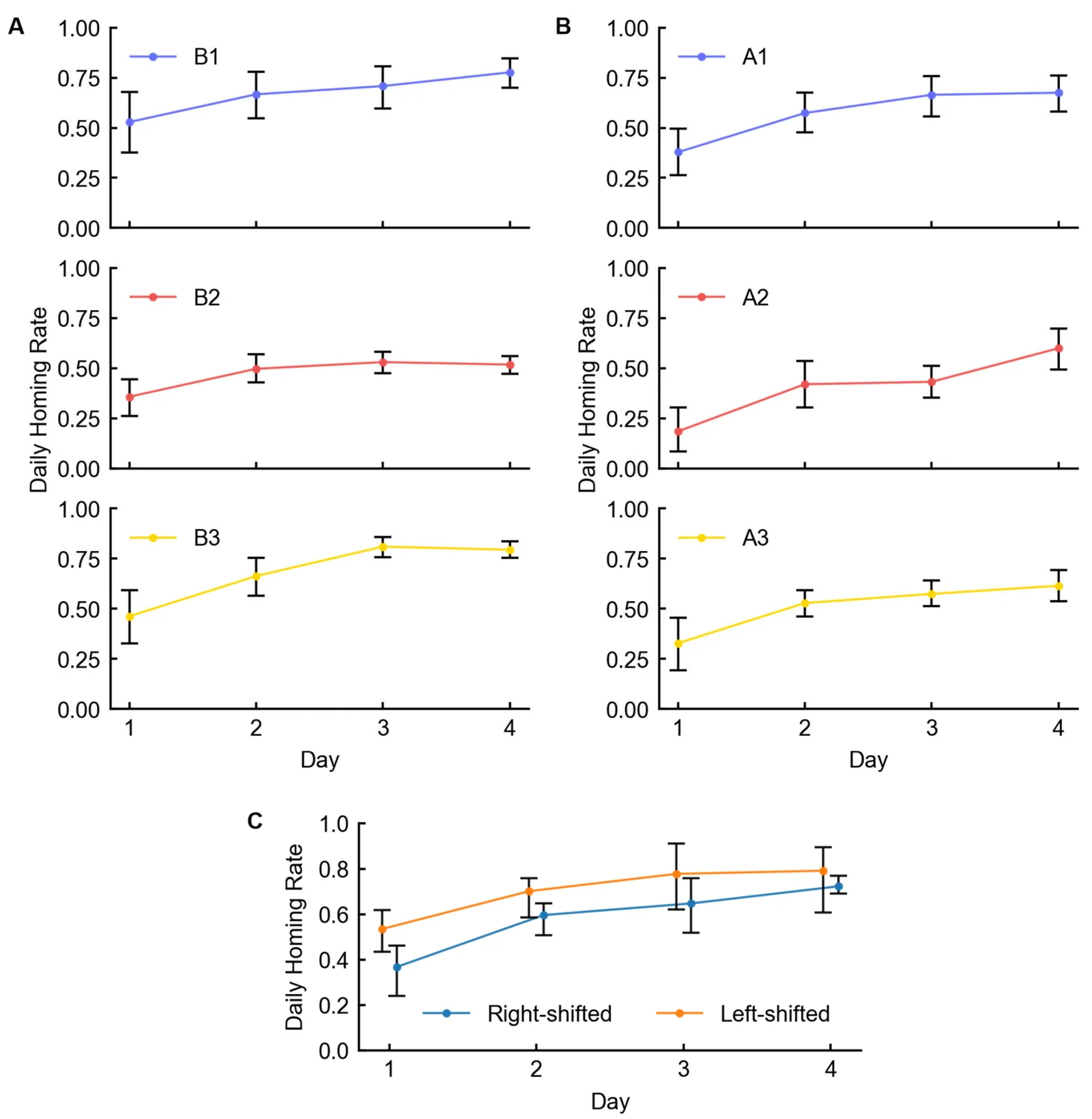
Dynamic Homing Rate of Groups A and B on days 1–4 after hive entrance displacement. (**A**) Right-shifted Group. (**B**) Left-shifted Group. (**C**) Comparative time-series curves under two different displacement patterns. Solid lines represent daily means, and error bars indicate 95% bootstrap confidence intervals, which accommodate the bounded [0, 1] nature and potential skewness of the data.

Figure 8A,B show the time-series changes in HR for Right-shifted and Left-shifted Group colonies during the 4-day period following hive entrance displacement. All six treated colonies displayed a similar dynamic pattern characterized by an initial rapid increase, followed by a slower increase (within-group: right-shifted *r* = 0.988, left-shifted *r* = 0.982; between-group *r* = 0.987; Figure 8C). To describe this 4-day dynamic pattern, the HR of six colonies was fitted using the asymptotic exponential model and the linear model. The asymptotic exponential trend provided a better fit than the linear trend (ΔAICc = 9.443; AICc = −52.723 for the asymptotic exponential model, AICc = −43.280 for the linear model). Therefore, the asymptotic model was used as a descriptive representation of the observed increase-and-plateau pattern. Colony-level asymptotic models converged for all six treated colonies (weighted $R^{2}$ : 0.938–0.991; Table 11). Across colonies, the estimated asymptotes *a* ranged from 0.539 to 0.930, and rates *k* ranged from 0.246 to 1.140. These parameters describe colony-level differences in the observed short-term HR dynamics.

#### 3.4.2. Time-Series Fitting Curve

**Figure 9:**
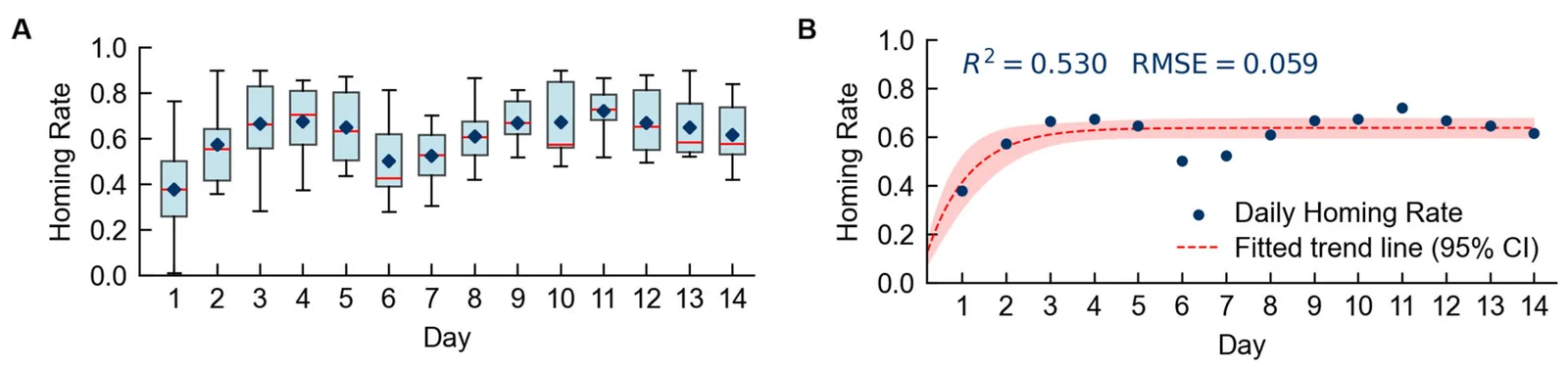
Long-term HR dynamics and model fitting for colony A1. (**A**) HR over 14 days post-displacement. Boxplots represent the distribution of HR values across daily observations, with the central line indicating the median and the box representing the interquartile range. Diamond markers indicate the daily mean HR. (**B**) Asymptotic exponential fitting curve of the HR. The red dashed line represents the fitted curve; the red shaded area denotes the 95% confidence interval; blue scatters indicate the daily mean HR values from the complete 14-day dataset.

The daily HR of colony A1 exhibited distinct temporal periods over the 14-day post-displacement period (Figure 9A). During Days 1–5, the daily mean HR showed a sharp increase that subsequently moderated, indicating an initial recovery following hive entrance displacement. On Day 6, the daily mean HR dropped sharply to 50.1%, followed by a recovery during Days 7–8 toward the level observed before the decline. Across Days 9–14, the daily mean HR fluctuated within a relatively stable range, with values ranging from 61.7% to 66.8% and peaking at 72% on Day 11. To mathematically characterize these dynamics, the complete 14-day dataset was fitted using an asymptotic exponential model as the primary analysis. This primary fit showed moderate performance ( $R^{2}$ = 0.530, RMSE = 0.059; Table 12), indicating that the complete HR trajectory was not fully captured by a simple asymptotic function. A sensitivity analysis was conducted to evaluate the influence of the Day 6–7 decline on model estimation. As shown in Table 12, the sensitivity analysis excluding Days 6–7 resulted in improved goodness-of-fit metrics, with $R^{2}$ increasing by 60.6% and RMSE decreasing by 45.8%. The estimated parameters showed relatively limited changes between the complete-data and sensitivity-analysis models. The asymptotic parameter *a* increased by 4.2% (from 0.637 to 0.664), and the rate constant ***k*** decreased by 9.3% (from 1.050 to 0.952). The estimated asymptotic level from the sensitivity analysis was 66.4%, compared with 63.7% from the complete-data model. Figure 9B presents the fitting curve based on the complete 14-day data.

## 4. Discussion

### 4.1. Conflicts in Homing Behavior Caused by Entrance Displacement

The observation of a bimodal distribution and region-specific paths reflects a dynamic transition from spatial memory based on the original entrance location to the exploration of the new location under an abrupt environmental change. Presumably, this is because bees first rely on old memories to return to the original hive entrance, and upon detecting the error, they initiate a local search to find the new one [25]. This search process aligns with the known mechanisms of initial and reorientation behavior, in which bees use the original entrance as a reference and obtain information about the location of the new hive entrance through sampling of the nearby space [54,55,56]. The heat map and trajectory map (Figure 6), coupled with the substantial reductions in HR (Figure 7), together qualitatively indicate that hive entrance displacement disrupts the orientation accuracy of honeybee colonies. During the baseline period, the variations among colonies were biologically negligible given that HR consistently exceeded 99%, establishing a uniform behavioral foundation prior to displacement. However, hive entrance displacement disrupted homing navigation, causing a substantial reduction in HR across treated groups. Although time-related environmental changes may also have affected HR, the Control Group showed a relatively small decrease in HR, suggesting that the larger declines observed in the treated groups were associated with hive entrance displacement. The observed difference between the Left-shifted and Right-shifted groups coincided with colony-level variation during the early post-displacement period. Video tracking logs showed that colonies A2 and A3 had no successful homing events at 07:00 on September 1, whereas such events were observed in the Left-shifted colonies at the same time. Given the small number of colonies in each group (*n* = 3) and the association between displacement direction and initial entrance-side position, the factors underlying the differences between the displacement groups cannot be determined from the present data. Overall, the substantial post-displacement reduction in HR was observed across the treated colonies. This substantial reduction in homing performance is consistent with the reliance of bees on previously established local spatial cues during entrance targeting. It indicates that the automated HR serves as a metric capable of quantifying the colony-level dynamics of short-range reorientation following hive entrance displacement.

### 4.2. Homing Rate Quantifies This Short-Range Reorientation Trend

HR links trajectory endpoints with the valid state of the hive entrance, thereby providing a continuously comparable colony-level metric for assessing entrance-targeting accuracy. The subsequent time-series analysis during the adjustment period shows how this automated metric captures the temporal dynamics of colony-level homing reorientation. The HR focuses on entrance selection rather than long-range homing capacity. It provides a unique lens to characterize the temporal dynamics of colony-level homing reorientation following changes in spatial cues near the entrance. Despite executing opposing physical vector alterations on treated groups, the colony-level trend in HR exhibited consistency. The striking synchronization in their post-displacement growth trends with a time-series correlation coefficient of 0.987 and localized within-group synchronization exceeding 0.98. Further, curve-fitting of these trends was conducted to mathematically capture and describe this synchronized dynamic. Compared with the linear model, the asymptotic exponential model provided a better quantitative description of the observed 4-day HR values following the hive entrance displacement. The initial increase observed during Days 1–2 was followed by a slower increase period toward the end of the observation period. Regarding the temporal scale, a potential constraint is that the multi-colony dataset spans only 4 days. However, honeybee orientation flights typically last 2 to 3 days [4,51,57]. Thus, a 4-day observation window primarily captures the early short-term changes in colony-level HR following hive entrance displacement. To further examine HR dynamics beyond the initial 4-day observation period, Colony A1 was analyzed as a long-term exploratory case study.

In the long-term observation of Colony A1, the initial asymptotic increase in daily HR was interrupted by a transient decline on Day 6. Day 6 coincided with the first clear day following prolonged cloudy conditions, suggesting that changes in environmental indicators may have contributed to this fluctuation. To further examine this possibility, we analyzed the associations between daily HR and illumination intensity (grayscale), temperature, and humidity in Colony A1. Illumination intensity showed a strong negative correlation with daily HR (*n* = 13; Spearman’s ρ = −0.632), whereas temperature and humidity showed only weak correlations (Supplementary Figures S2 and S3). This provides additional evidence that changes in light conditions were temporally associated with the transient decline in HR (Supplementary Figure S4). However, this correlation does not provide sufficient evidence for a causal relationship, given that the analysis was based on a single colony A1 with a limited sample size. Therefore, the exclusion of Days 6–7 was used as a sensitivity analysis to evaluate how the transient decline influenced model fit and parameter estimation. The subsequent adaptation in HR from Days 7–9 reflects that the decline was transient rather than persistent. Excluding the data from Days 6–7 substantially enhanced the goodness of fit ( $R^{2}$ , increased from 0.530 to 0.851) and reduced estimation error (RMSE decreased by 45.8%). More importantly, the estimated parameters (a and k) differed by less than 10% between the full dataset and the sensitivity analysis. This suggests that the transient fluctuation had a strong influence on goodness of fit but a comparatively limited influence on the estimated asymptotic level and rate parameter. It did not alter the overall colony-level HR trend after hive entrance displacement, which consisted of an initial adaptation period and subsequent stabilization.

The model provides a mathematical interpretation of the observed behavioral shift, in which the recovery of entrance-targeting accuracy can be described by an asymptotic exponential adaptation curve. Under the environmental stress of hive entrance displacement, the parameter *k* (*k* = 1.050) described the estimated rate of HR recovery in Colony A1 during the initial increase period. The estimated asymptotic level *a* (*a* = 0.637) derived from the model can be interpreted as the plateau observed within the 14-day period following nest entrance displacement. These parameters describe the estimated rate and extent of colony-level HR change together, providing quantitative indicators for characterizing behavioral responses to entrance displacement. Additionally, the plateau below 100% HR indicates that the colony-level entrance-targeting outcome reached a stable level rather than complete recovery. It may be attributed to the combined effects of multiple sources, including individual variation in learning performance, repeated decision-making during homing flights, and environmental fluctuations during the observation period. First, some bees with weaker learning abilities may not fully adapt to the cues of the new entrance location. Similarly, Tait found that individuals showed differences in visual and olfactory learning and affected group performance [58,59]. The consistency of individual differences in different learning tasks [50,51,52,53] indicates that learning ability is a stable trait across contexts [60]. Second, the honeybee colony consists of bees with different ages and task states, which may generate different responses to environmental changes and influence colony-level behavioral outcomes [61,62]. Third, the reorientation may involve repeated approaches and hovering near entrances during the selection of the target entrance [2,25,63], and tracking-related ID continuity errors may further contribute to variability in colony-level homing outcomes. Without individual-level tracking and controlled environmental conditions, the specific factors underlying incomplete adaptation of homing redirection during the 14-day observation period cannot be further resolved.

### 4.3. Reliability and Validation of the Homing Rate

To evaluate the methodological reliability of the proposed digital phenotyping pipeline, we performed a multi-tiered validation comprising algorithmic error analysis, manual ground-truth comparison, and temporal bias evaluation. Rather than developing new detection or tracking algorithms, this study focused on adapting mature detection and tracking algorithms to the high-density, occluded, and semi-natural apiary environment. In the baseline comparison of YOLO11 configurations (Table 5), YOLO11m exhibited optimal adaptation to the crowded and occluded hive entrance scene, significantly outperforming the ultra-lightweight variants (YOLO11n and YOLO11s) with a Precision of 0.916, a Recall of 0.858, and an mAP@0.5:0.95 of 0.572. Crucially, its mAP@50 (0.920) converged with the performance of large-scale alternatives (YOLO11l and YOLO11x) while maintaining a high inference throughput (27.91 fps), demonstrating its capability for sustaining long-term continuous tracking. In the evaluation of candidate trackers based on YOLO11m, OC-SORT and ByteTrack showed different performance advantages. DeepSORT and StrongSORT showed intermediate performance across the evaluated metrics. ByteTrack achieved the highest IDF1 (0.685) and the lowest IDsw (8) and FN (243), indicating great identity maintenance. Although OC-SORT was slightly inferior to ByteTrack in identity maintenance (IDsw = 12, IDF1 = 0.642), it maintained the second-lowest FN (556) while achieving the highest MOTA and MOTP and the lowest FP. The MOT algorithm selection considered overall performance across several tracking metrics, including identity maintenance, tracking accuracy, FP, and FN. Long-term individual identity maintenance is important for trajectory reconstruction, but OC-SORT provided a more favorable overall performance across those metrics, all of which can affect HR calculation. Therefore, the combined pipeline of YOLO11m and OC-SORT is a well-suited engineering decision aligned with this monitoring area, providing a practical tool for the subsequent quantitative analysis of homing behavior. Next, the accuracy of the derived HR measurements must also be evaluated under the actual experimental conditions.

Manual validation using the 2025 experimental videos revealed different error characteristics between the two periods. During baseline, the low RMSE, near-zero mean bias (MB = −0.002), and narrow 95% LoA indicated close agreement between automated and manual measurements. In contrast, adjustment showed a higher RMSE, wider 95% LoA, and a positive mean bias (MB = 0.087), indicating greater measurement error and a tendency to overestimate HR. This difference partly reflects the definition of HR itself. During baseline, very few trajectories terminated at the inactive entrance position, causing HR values to cluster near 1 and limiting the influence of tracking errors. During adjustment, trajectories were distributed between the new and former entrance positions, increasing HR variability and the potential influence of tracking errors on HR estimates. The positive bias further indicates that automated HR tended to overestimate the proportion of trajectories terminating at the valid entrance, thereby reducing the observed decline in HR. Nevertheless, HR still showed a substantial reduction after displacement. The linear relationship (*r* = 0.963) between automated and manual HR, with the quantified measurement error, indicates that the automated HR captured the overall variation in manual HR. These results support the use of automated HR for comparing relative changes in colony-level homing behavior under the experimental conditions of this study. This positive bias also affects the model parameters estimated from adjustment-period HR values. Because automated HR tends to be higher than manual HR, the asymptotic level *a* may be overestimated. The bias is smaller at lower HR values and greater at higher HR values (Figure 4A). This error pattern may increase the estimated rate parameter *k*, making adaptation to reach the asymptotic level more rapidly. Therefore, the fitted parameters and adaptation estimates should be interpreted as descriptive estimates based on automated HR. In addition, this end-to-end manual comparison incorporates the combined effects of detection, tracking, trajectory filtering, and endpoint classification on the final HR estimates, but it does not isolate the individual contributions of identity switches, short-trajectory exclusion, or the displacement-jump threshold. These tracking-related sources of error therefore remain a limitation of the present analysis.

To ensure the validity of the behavioral conclusions, potential experimental effects induced by the time span were considered in the quantitative test. The Type III tests of fixed effects revealed a significant interaction effect between Right-shifted Group and period (Table 8). Subsequent period contrast analyses (Table 9) indicated that the comparisons across periods for the blank Control Group showed slight differences (RD = −0.13%), with the confidence intervals overlapping substantially. Combined with the between-group analysis during the adjustment period, these results demonstrated that temporal factors and environmental fluctuations were insufficient to explain the substantial reduction in HR observed in the treated groups. This confirms the qualitative observation that no differences in hive entrance clustering emerged between the heatmaps of C1 in the Control Group before and after the displacement. Conversely, both Right-shifted and Left-shifted Groups experienced a highly significant, precipitous decline in HR following the intervention (Table 9). These data comparisons demonstrate that the decline in HR was not driven by temporal bias or environmental fluctuations. Rather, hive entrance displacement was the primary factor causing colony disorientation. This cross-validation indicates that the observed dynamics in the automated HR metric reflect the biological signal of honeybees’ homing entrance-targeting conflict and adaptation outlined in our introduction and were not solely attributable to systematic errors introduced by computer vision tracking and time-varying environmental factors.

### 4.4. Limitations, Future Prospects, and Applications

Because the current tracking method reconstructs honeybee trajectories only within individual video segments and does not provide persistent individual identification across time, this study cannot directly determine how individual behavioral variation contributes to colony-level HR dynamics and their asymptotic characteristics. Future work could integrate the automated pipeline with individual marking identification to enable longitudinal tracking of the same bees. When combined with marking of known-age individuals, this approach could further examine how age, experience, and individual behavioral variation relate to colony-level HR dynamics. Such analyses would help clarify the individual-level basis of the observed colony-level patterns. Meanwhile, the asymptotic level parameter *a* and rate parameter *k* may serve as candidate descriptors of colony-level HR dynamics. However, the present study was conducted in a single apiary with nine colonies, a 30-cm entrance displacement, and long-term analysis centered on Colony A1. Therefore, the robustness and applicability of our findings require validation across larger colony samples, multiple apiaries, different displacement distances, seasons, and environmental conditions. In addition, although hive placement was randomized and camera orientation was standardized across colonies, displacement direction remained associated with the initial entrance-side position. Therefore, their independent effects could not be separated, and differences between the Right-shifted and Left-shifted Groups should be interpreted cautiously. Future experiments should apply leftward and rightward displacements from the same initial entrance-side position to evaluate potential lateral preferences in colony-level homing responses.

Beyond behavioral ecology, the proposed quantification pipeline may also be used to evaluate behavioral responses to hive-entrance management interventions. Practices such as using two-entrance hives or dynamically adjusting hive entrances alter the local spatial cues encountered during the final stage of homing and may therefore affect entrance targeting. The asymptotic increase in HR during the first 3 days, the estimated asymptotic level of 63.7%, and the recovery following the transient decline on Day 6 were descriptive observations from Colony A1 over the 14-day period. Nevertheless, these results indicate that temporal changes in HR can quantitatively describe changes in entrance-targeting outcomes over time, providing an automated alternative to conventional visual assessment.

In future apiaries equipped with long-term continuous computer vision monitoring, this approach could be extended to more colonies and different entrance-management conditions to evaluate the repeatability of colony-level HR dynamics and associated model parameters. Continuous monitoring after entrance adjustment could further quantify the time course from the initial disturbance to gradual recovery and stabilization of entrance-targeting behavior, and allow comparisons of recovery rate and stabilized level among colonies or management conditions. With validation from larger datasets, these temporal parameters could be used to estimate the recovery timescale following entrance adjustment under specific management conditions, thereby providing quantitative guidance for post-adjustment observation periods, the timing of subsequent operations, and management optimization. Thus, this study provides a methodological framework for continuously monitoring colony-level entrance-targeting behavior and quantifying recovery dynamics following hive-entrance adjustment.

## 5. Conclusions

This study established a non-invasive automated pipeline integrating YOLO11m-based detection, OC-SORT tracking, and the Homing Rate (HR) to quantify colony-level short-range homing reorientation following hive entrance displacement. HR links trajectory endpoints with the valid entrance state, providing a continuously comparable colony-level measure of entrance-targeting accuracy. Horizontal entrance displacement caused a substantial reduction in HR in the treated colonies, followed by a gradual increase toward a relatively stable level. The asymptotic exponential model provided a descriptive representation of this temporal adaptation pattern. The 14-day observation of Colony A1 further showed an early increase, transient decline, and subsequent recovery toward a relatively stable level, which remained below 100% and differed from the near-complete levels observed before displacement and in the Control Group. The fitted parameters *a* and *k* may serve as candidate descriptors of the extent and rate of colony-level HR adaptation dynamics, although their repeatability and applicability require further validation across larger colony samples and broader experimental conditions. Overall, this study provides a pipeline for continuously monitoring colony-level entrance-targeting behavior and quantifying adaptation dynamics following hive entrance displacement.

## Figures and Tables

**Table 1:** Hive entrance displacement configurations by group.

Group	Initial Position	Displacement	Final Position
Right-shifted	Left	Rightward 30 cm	Right
Left-shifted	Right	Leftward 30 cm	Left
Control	Left/Right	-	Left/Right

**Table 2:** Details of the dataset used for tracking and evaluation.

ID	Duration (s)	Bounding Box Count	Frames	Average Density (Bees/Frame)
1	10	4095	250	16.4
2	10	1757	250	7.0
3	10	1535	250	6.1
4	10	2837	250	11.3

**Table 3:** Summary of data volume across experimental periods.

Period	Colonies	Date	Raw Video	Analytical Sample
Baseline	Right-shifted Group, Left-shifted Group, Control Group	1–2 August	216 h	216 min
Adjustment	Right-shifted Group, Left-shifted Group, Control Group	1–2 September	216 h	216 min
Post-displacement	Right-shifted Group, Left-shifted Group	1–4 September	288 h	288 min
Long-term tracking	Colony A1	1–14 September	168 h	168 min

**Table 4:** Classification criteria for homing trajectory directions.

**Direction**	Range	Meaning
Upward	$\frac{\pi }{4}\;<\;\theta \;\leq \;\frac{3\pi }{4}$	Clear homing-intent, direct radial approach toward the hive entrance.
Leftward	$\frac{3\pi }{4}\;<\;\theta \;\leq \;\frac{5\pi }{4}$	Horizontal leftward translation, lateral position adjustment.
Downward	$\frac{5\pi }{4}\;<\;\theta \;\leq \;\frac{7\pi }{4}$	Moving away from the hive entrance, obstacle avoidance, disturbance, or reorientation.
Rightward	$\frac{7\pi }{4}\;<\;\theta \;\leq \;2\pi ,\;0\;<\;\theta \;\leq \;\frac{\pi }{4}$	Horizontal rightward translation, lateral position adjustment.

**Table 5:** Results of YOLO11 models in the bee detection task.

Metrics	YOLO11n	YOLO11s	YOLO11m	YOLO11l	YOLO11x
Best epoch	214	228	198	204	225
Precision (%)	89.0	90.6	**91.6**	89.9	90.5
Recall (%)	82.9	85.9	85.8	**87.9**	87.5
mAP@50 (%)	89.5	91.5	92	**92.5**	92.3
mAP@0.5:0.95 (%)	53.2	55.8	**57.2**	56.9	57.1
Speed (fps)	**27.97**	22.69	27.91	21.50	24.73

Bold values indicate the best performance for each metric, and underlined values indicate the second-best performance in Table 5 and Table 6.

**Table 6:** Results of different MOT models in the tracking evaluation task.

Metrics	ByteTrack	StrongSORT	DeepSORT	OC-SORT
IDF1 (%) ^1^	**68.5**	38.8	47.3	64.2
FP ^2^	1167	779	662	**278**
FN ^3^	**243**	980	929	556
IDsw ^4^	**8**	99	64	12
MOTA (%) ^5^	52.4	54.7	60.3	**67.2**
MOTP (%) ^6^	70.2	70.7	71.3	**72.9**

^1^ IDF1, ID F1-score; ^2^ FP, false positives; ^3^ FN, false negatives; ^4^ IDsw, ID switches; ^5^ MOTA, multiple object tracking accuracy; ^6^ MOTP, multiple object tracking precision. Higher values for IDF1, MOTA, and MOTP, and lower values for FP, FN, and IDsw indicate better performance.

**Table 7:** Period-level validation between automated and manual HR measurements.

Period	*n*	$R^{2}$	RMSE	MB	95% LoA
Baseline	12	0.780	0.010	−0.002	−0.022–0.017
Adjustment	24	0.825	0.122	0.087	−0.084–0.258

**Table 8:** Type III Wald χ^2^ test results of GLMM.

Effect	*χ* ^2^	df	*p*
Group	6.93	2	0.031
Period	0.58	1	0.448
Group × Period	42.12	2	<0.001

**Table 9:** Within-group pairwise comparisons of HR across periods.

Group	Contrast	RD ^1^	OR ^2^	95% CI ^3^	*p* ^4^
Right-shifted	Adjustment–Baseline	−50.36%	0.008	(0.006, 0.011)	<0.001
Left-shifted	Adjustment–Baseline	−40.89%	0.010	(0.007, 0.015)	<0.001
Control	Adjustment–Baseline	−0.13%	0.612	(0.173, 2.173)	0.448

^1^ RD, rate difference; ^2^ OR, odds ratio. OR < 1 indicates lower odds relative to the reference group (Baseline period in Table 9; Control Group in Table 10); ^3^ CI, confidence interval. ^4^ *p*-values were adjusted for multiple comparisons using the Tukey method.

**Table 10:** Within-period pairwise comparisons of HR among treated groups.

Period	Contrast	RD	OR	95% CI	*p*
Baseline	Right-shifted–Control	−0.68%	0.236	(0.065, 0.855)	0.023
	Left-shifted–Control	−0.53%	0.276	(0.076, 1.002)	0.051
	Right-shifted–Left-shifted	−0.15%	0.856	(0.439, 1.668)	0.849
Adjustment	Right-shifted–Control	−50.91%	0.003	(0.001, 0.009)	<0.001
	Left-shifted–Control	−41.29%	0.005	(0.002, 0.013)	<0.001
	Right-shifted–Left-shifted	−9.62%	0.649	(0.450, 0.936)	0.015

**Table 11:** Colony-level parameter estimates from the asymptotic exponential model.

Colony	*a* ^1^	*k* ^2^	$R^{2}$ ^3^	*RMSE*
A1	0.721	0.774	0.991	0.0114
A2	0.930	0.246	0.938	0.0416
A3	0.651	0.746	0.984	0.0145
B1	0.761	1.120	0.943	0.0207
B2	0.539	1.140	0.972	0.0119
B3	0.854	0.795	0.965	0.0228

^1^ *a*, asymptotic parameter; ^2^ *k*, rate parameter; ^3^ $R^{2}$ , coefficient of determination. The weighted $R^{2}$ values are descriptive indicators only and should be interpreted with caution given the limited number of daily time points (*n* = 4).

**Table 12:** Sensitivity analysis for daily homing rate models in colony A1.

Dataset	n	*a*	*k*	$R^{2}$	*RMSE*
Complete period (1–14 September)	14	0.637	1.050	0.530	0.059
Sensitivity check (Excluding 6–7 September)	12	0.664	0.952	0.851	0.032
Percentage shift	-	+4.2%	−9.3%	+60.6%	−45.8%

## Data Availability

The data supporting the findings of this study are available in the article and its Supplementary Materials. Additional data are available from the corresponding authors upon reasonable request.

## Supplementary Materials

The following supporting information can be downloaded at: https://www.mdpi.com/article/10.3390/insects17090944/s1, Figure S1: Schematic layout of hive placement in the apiary; Table S1: Parameters of the smart hive; Table S2: Illumination distribution of the bee detection dataset; Note S1: Rationale for Selection of Candidate Multi-Object Tracking (MOT) Algorithms; Note S2: Cartesian Coordinate System Establishment and Trajectory Angle Calculation Formulae; Figure S2: Spearman correlations between Homing Rate and environmental variables; Figure S3: Scatterplot matrix of Homing Rate and environmental variables; Figure S4: Daily variation in illumination intensity during the observation period.
