# Supplementary material for: Machine Vision-Based Quantification of Colony-Level Homing Adaptation in Apis mellifera Following Hive Entrance Displacement
Source: Insects. 2026 Sep 10;17(9):944. doi: 10.3390/insects17090944 (PMC13607679; doi:10.3390/insects17090944)
Supplement: Supplementary file 1 [file insects-17-00944-s001.zip › insects-4509433-supplementary.pdf]

Supplementary Material

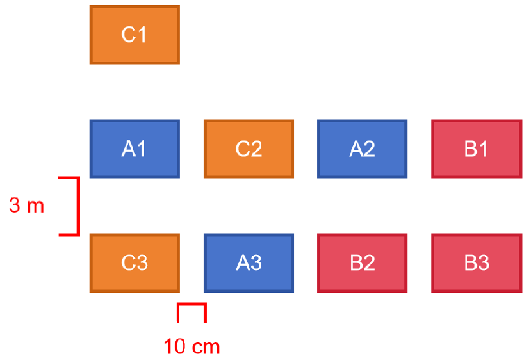

Figure S1. Schematic layout of hive placement in the apiary. The figure shows the relative spatial arrangement of the experimental hives. Adjacent hives within each row were spaced approximately 10 cm apart, and the distance between rows was approximately 3 m.

Table S1. Parameters of the smart hive

Parameter	Value
Hive dimensions (internal)	37 × 47 × 26 cm
Entrance dimensions (×2)	48 × 7 mm
Camera	Ezviz C8W
Data storage	microSD card
Video frame rate	25 FPS
Image resolution	2560 x 1440 P

Table S2. Illumination distribution of the bee detection dataset.

Illumination Level	Range	Image Count	Percentage (%)
Low	0-85	10	1.00
Medium	85-170	895	89.23
High	170-255	98	9.77
Total	54.51-216.14	1003	100

Note: Image brightness is expressed as the mean grayscale intensity. The classification is based on the equal-width binning method.

Note S1. Rationale for Selection of Candidate Multi-Object Tracking (MOT) Algorithms.

Under the Tracking-by-Detection paradigm, multi-object tracking accuracy in dense insect monitoring relies heavily on the robustness of bounding-box association. In this study, the classic SORT algorithm serves as the baseline architecture for long-term trajectory extraction, utilizing a Kalman filter for state prediction and the Hungarian algorithm for optimal Intersection-over-Union (IoU) cost matrix assignment. However, the highly non-linear movement profiles of honeybees within the near-entrance area frequently violate the linear assumptions of standard Kalman filtering, leading to prediction drift and frequent identity switches. To explore how different technical mechanisms optimize trajectory continuity under these biological constraints, four representative tracking algorithms were selected for comparative evaluation:

1. DeepSORT and StrongSORT represent an appearance-assisted approach.

DeepSORT [41] incorporates appearance feature re-identification (Re-ID) to construct individual feature profiles, leveraging appearance dissimilarity to assist spatial matching after brief target occlusions, thereby mitigating ID switches caused by frequent object crossings. StrongSORT [42] inherits and expands upon DeepSORT's tracking paradigm of combining appearance Re-ID with motion state prediction. Specifically, it refines the Re-ID extraction network and appearance state updating mechanism, optimizes the weight assignment and normalized fusion algorithm for the appearance and motion cost matrices, and introduces Appearance-Free Link (AFLink) along with Guided Motion Interpolation (HMI) for trajectory post-processing and tracklet reconnection. This mechanism tests whether appearance features can resolve identity fragmentation and maintain tracklet continuity when bees frequently cross paths or undergo short-term occlusions.

2. ByteTrack represents an association-logic refinement approach.

ByteTrack [45] implements a two-stage association strategy that preserves and processes low-confidence detection boxes rather than discarding them. This approach investigates whether retaining low-score bounding boxes can prevent trajectory termination when honeybees experience partial body occlusion or severe posture deformation near the entrance.

3. OC-SORT represents a motion-centric error-correction approach.

OC-SORT [46] employs velocity direction compensation and observation-centric heuristics to directly rectify Kalman filter prediction drift. This mechanism tests whether motion-centric adjustments can effectively counteract the errors introduced by erratic and non-linear honeybee flight trajectories without relying heavily on appearance features.

Note S2. Cartesian Coordinate System Establishment and Trajectory Angle Calculation Formulae

To discretize continuous spatial tracking data into discrete directional categories including Upward, Leftward, Downward, and Rightward, a localized Cartesian coordinate system was defined for each homing-intent trajectory.For coordinate origin and axis orientation, a Cartesian coordinate system is established with the starting point of the trajectory (x_{start},y_{start}) as the origin (0, 0). The horizontal rightward direction is defined as 0, and the vertical upward direction toward the hive is defined as $\pi/2$. For trajectory vector angle calculation, the angle θ represents the orientation of the displacement vector connecting the starting point (x_{start},y_{start}) and the endpoint (x_{end},y_{end}). Taking into account the standard image coordinate system where the vertical y-axis originates from the top-left corner, the angle θ is derived as follows:

$$\Delta x = x_{end} - x_{start},$$

$$\Delta y = -(y_{end} - y_{start}),$$

$$\theta = atan2(\Delta y, \Delta x)\ (mod\ 2\pi).$$

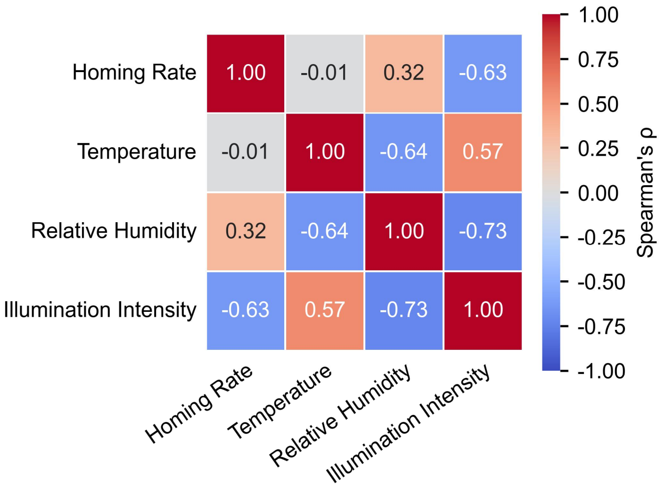

Figure S2. Spearman correlations between homing rate and environmental variables. Heatmap showing Spearman's rank correlation coefficients among daily mean homing rate, temperature, relative humidity, and illumination intensity (n = 13 days).

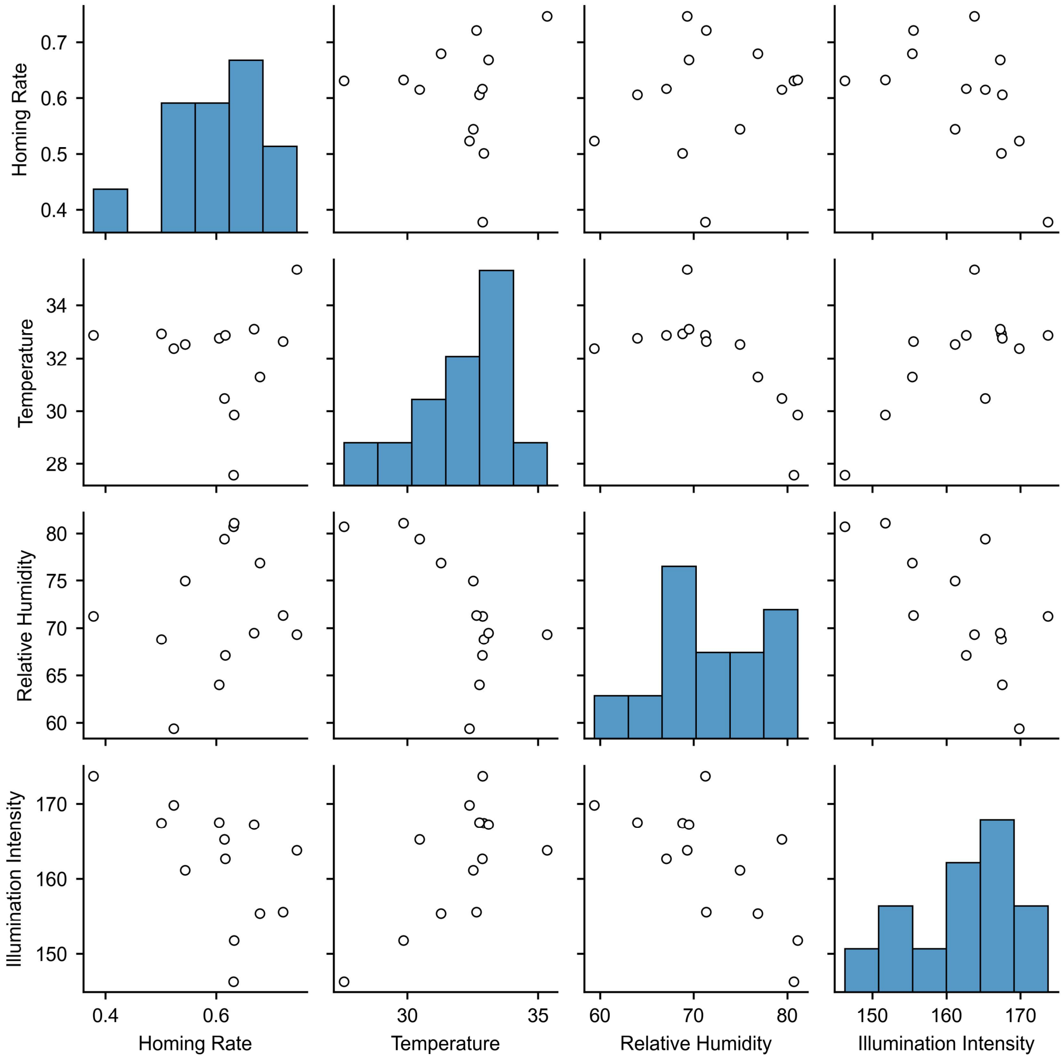

Figure S3. Scatterplot matrix of Homing Rate and environmental variables. Scatterplot matrix showing daily mean homing rate, temperature, relative humidity, and illumination intensity (n = 13 days). Data for 3 September were excluded because temperature measurements were unavailable due to equipment failure.

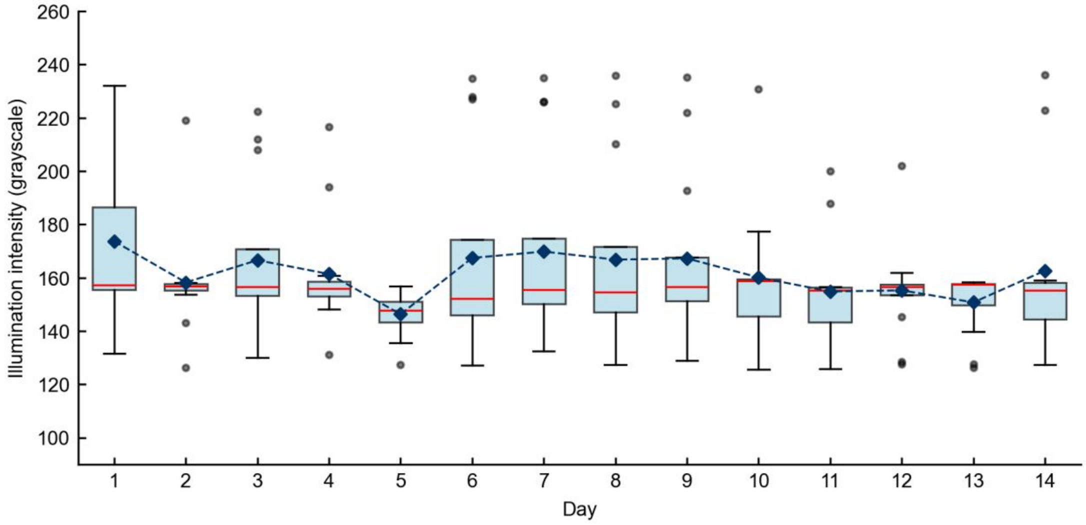

Figure S4. Daily variation in illumination intensity during the observation period. Boxplots show the distribution of illumination intensity (grayscale) for each day. Red horizontal lines indicate medians; diamonds indicate daily means; whiskers extend to values within 1.5 IQR; circles indicate outliers; dashed lines connect daily mean diamonds.
